# Patient-reported outcome measure and its application in patients with stroke: item response theory

**DOI:** 10.1136/svn-2024-003166

**Published:** 2024-05-30

**Authors:** Jia Ma, Jinma Ren, Joseph C Cappelleri

**Affiliations:** 1 Statistical Research & Data Science Center, Pfizer, Groton, Connecticut, USA; 2 Statistical Research & Data Science Center, Pfizer, Collegeville, Pennsylvania, USA

**Keywords:** Statistics, Stroke, Technology

## Introduction

 In recent years, the concept of patient-centred care in the healthcare industry has gained increasing focus and recognition on the demand for patient-reported outcome (PRO) measures. A PRO is defined as ‘any report of the status of a patient’s health condition that comes directly from the patient, without interpretation of the patient’s response by a clinician or anyone else’.^1^ PRO measures are employed to capture the patient status with a whole array of multidimensional health attributes collectively including symptoms, functions and general health in a variety of concepts, such as pain, fatigue, depression, aspect of well-being and quality of life.^2^ PROs play important roles as endpoints in clinical trials to evaluate the efficacy, benefit and risks of drug and other medical products from the patient’s perspective.

The US Food and Drug Administration (FDA) roadmap requires considering fit-for-purpose clinical outcome assessments (COAs)—using an existing, a modified or a newly developed measure.^3^ Traditionally, the questionnaires are developed by classic test theory (CTT) with fixed (static) form that all the patients are being asked to answer the same set of questions. For instance, the legacy PRO measures for stroke include Stroke Specific Quality Of Life scale (SS-QOL), Stroke Impact Scale (SIS), Stroke-adapted 30-item version of the Sickness Impact Profile (SA-SIP30), SATIS-Stroke and so on. Later, the application of item response theory (IRT) has been adapted to PRO measures from educational measurement to medical applications including stroke. In practice, there are diverse IRT-based PRO measures that can efficiently help identify what is important to patients, including fixed, customised or computerised adaptive testing (CAT, a computer-based test adapting to the patient’s severity/ability level) forms. However, the advantages of IRT have not been solely understood by clinicians and clinical researchers.

## Trend of using IRT-based PROs from industry guidance

The FDA has issued a PRO guidance for industry for incorporating patient input in clinical trials since 2009.^1^ More recently, the 2022 FDA draft guidance on selecting, developing or modifying fit-for-purpose COAs has given consideration on the important details for psychometric methods on COA development, including IRT models (eg, Rasch, graded response and partial credit models), the implementation of IRT assumptions, sample size and model fit, as well as the scoring approach for CAT.^3^

The 2023 FDA draft guidance addresses the methodologies, standards and technologies for COAs in clinical trials.^4^ In this guidance, a well-justified CAT design was considered as an option for collecting patients’ scores. Later in the year, the FDA guidance for technical specification using IRT for COA data has been published.^5^ Given the timeliness and relevance of this specific guidance on IRT, and with the rapid development on information technology (eg, electronic COA), the measure of PRO endpoints is becoming a viable way to capture a patient’s direct insight through the efficient application of IRT.

## IRT for PROs

IRT is also known as latent trait theory in psychometrics. It is based on mathematical models behind PROs that present the relationship of an individual’s response to each item and the latent trait of the questionnaire measures. The probability of individuals’ responses to the item is given to a mathematical function called item response function, which relates the characteristics of the item and individuals to the probability of endorsing a given response to the item.^6^ Consequently, the parameter estimation consists of item and person parameters. Using graded response model as an example, item parameters characterise an item, including the numerical values on (1) the parameter *a* to differentiate among patients at various levels to the trait; and (2) the parameter *b* represents the severity of the condition on the item measures, while person parameters represent the threshold (*ө*) where the individual stands on the underlying concept of interest, such as pain, fatigue or depression as the unobservable trait.

The values for parameters are estimated through statistical methods, such as maximum likelihood and Bayesian methods, and goodness-of-fit tests are used to evaluate the appropriateness of the IRT model with respect to the empirical study data. Different from reliability in CTT, reliability in IRT is conceptualised as information that reflects the precision of the measurement across the level of underlying trait and is independent of the sample administered. CTT assumes that an item assesses the trait with the same precision for everyone, while IRT can assess individuals’ score more accurately based on the person’s performance.^7^ Nevertheless, as a model-based method, IRT requires a large sample size, hypothetical assumptions (unidimensionality, local independence of items and measurement invariance), and acceptable item and model fit (ie, fit as consistent with the empirical data). Failure to meet these requirements may compromise the integrity of the evidence from IRT-based PRO assessments.

## Item bank and automated test assembly

A PRO measure contains a set of items in one or multiple domains. The approach of applying IRT to create a questionnaire is item selection covering target aspects of a patient’s conditions.^8^ This requires a number of well-developed items stored in a database, named an item bank. An item bank is the place for storing items as well as their specifications and administration requests. These specifications, originally described in verbal statements, are translated into standard expression that is recognised by computer programs for select items to assemble the questionnaire, called automated test assembly (ATA).^9 10^ For a fixed form, all the items are selected from the item bank at the same time to achieve the prespecified criterion such as a specified number of items and specified level of precision. For CAT, after the initial item is answered, each additional item is assigned based on the performance of the current item until the prespecified criterion has been achieved. With the significant development in computer technology from 1980s, a great amount of research has been done in applying mathematical programming techniques into optimisation^11 12^ and test assembly algorithm,^13 14^ which takes the test development to a new chapter for ATA.

## Application of IRT-based PROs in stroke

During the past two decades, IRT-based PROs have been developed in diverse therapeutic areas. A well-known example is the Patient-Reported Outcomes Measurement Information System (PROMIS) initiative from the National Institutes of Health that developed a series of domain-specific measures in multiple chronic conditions including stroke.^15^ The significance of PROMIS is building item banks for different domains to support constructing fit-for-purpose PROs in fixed form, customised form and CAT. It provides standard metric on t-score tables with the mean of 50 and the SD of 10 for comparing the patients’ score of quality of life with the US general population and several populations with specific disease.

Another IRT-based PRO example is the Neuro-QoL (Quality of Life in Neurological Disorders), which evaluates and monitors the physical, mental and social effects experienced by adults and children living with neurological conditions including stroke.^16^ Each domain (eg, physical function) includes item banks, and each item bank consists of multiple items. As shown in table 1, the item bank of mobility (Neuro-QoL Short Form V.1.0–Lower Extremity Function) has eight items. Each item is measured by a 5-point Likert scale from ‘1=unable to do’ to ‘5=without any difficulty’ with a higher score indicating better mobility.^17^ These examples have clearly drawn the trend of PRO development and application using IRT, although IRT-based PROs in clinical trials are still in development and have not matured yet.

**Table 1:** An example of Neuro-QoL Short Form V.1.0–Lower Extremity Function

Question	Without any difficulty (5)	With a little difficulty (4)	With some difficulty (3)	With much difficulty (2)	Unable to do (1)
Are you able to get on and off the toilet?					
Are you able to step up and down curbs?					
Are you able to get in and out of a car?					
Are you able to get out of bed into a chair?					
Are you able to push open a heavy door?					
Are you able to run errands and shop?					
Are you able to get up off the floor from lying on your back without help?					
Are you able to go for a walk for at least 15 min?					

Note: Neuro-QoL measures are copyrighted. Commercial users must seek permission to use, reproduce, or distribute measures. Integration into proprietary technology requires written permission.

Neuro-QoLQuality of Life in Neurological Disorders

Potential advantages of using IRT-based PRO measurement systems are not only for the ability to capture multiple domains of importance to patients, but also increasing the efficiency and reliability of data collection, increasing statistical power and improving sensitivity to change (responsiveness).^18^ A recent systematic literature review has described the use of outcomes of PROMIS measures in clinical studies in populations with stroke, which suggests that the PROMIS measures could be relevant from a patient’s perspective, covering the International Classification of Functioning, Disability, and Health core set domains for patients with stroke.^19^ Most of studies identified in that review were published after the international standard set of PRO measures after stroke was developed in 2016.^20^

## The future of IRT-based PROs

The application of IRT has shown rapid growth for the need of PRO measures in clinical trials. IRT had experienced many years of development in education measurement fields before being applied to varying degrees of success in the healthcare industry and medical field. In principle, the advantages of IRT have become understandable and apparent in PRO measures and have been formally acknowledged in regulatory guidance to the healthcare sponsors. When the methods are adapted to clinical research, adjustments from the nature of the content should be made, especially for meeting the high requirements of regulatory submission. Although the well-defined and validated legacy measures in stroke need to be continually considered in practice, IRT-based PRO measures should also be explored and taken into account as the merit of IRT has become widely recognised. Nevertheless, it is worth noting that PRO measures may not be applicable to some patients and other types of COAs (eg, clinician or observer-reported outcomes) should be used if patients who had a stroke have cognitive impairment.
